# Optimized Use of Low-Depth Genotyping-by-Sequencing for Genomic Prediction Among Multi-Parental Family Pools and Single Plants in Perennial Ryegrass (*Lolium perenne* L.)

**DOI:** 10.3389/fpls.2018.00369

**Published:** 2018-03-21

**Authors:** Fabio Cericola, Ingo Lenk, Dario Fè, Stephen Byrne, Christian S. Jensen, Morten G. Pedersen, Torben Asp, Just Jensen, Luc Janss

**Affiliations:** ^1^Department of Molecular Biology and Genetics, Center for Quantitative Genetics and Genomics, Aarhus University, Tjele, Denmark; ^2^DLF Seeds A/S, Research Division, Store Heddinge, Denmark; ^3^Department of Molecular Biology and Genetics, Crop Genetics and Biotechnology, Aarhus University, Slagelse, Denmark; ^4^Teagasc, Department of Crop Science, Carlow, Ireland

**Keywords:** Perennial ryegrass, sequencing depth, genomic relationship matrix, family pools, genotyping by sequencing, missing value imputation, genomic prediction

## Abstract

Ryegrass single plants, bi-parental family pools, and multi-parental family pools are often genotyped, based on allele-frequencies using genotyping-by-sequencing (GBS) assays. GBS assays can be performed at low-coverage depth to reduce costs. However, reducing the coverage depth leads to a higher proportion of missing data, and leads to a reduction in accuracy when identifying the allele-frequency at each locus. As a consequence of the latter, genomic relationship matrices (GRMs) will be biased. This bias in GRMs affects variance estimates and the accuracy of GBLUP for genomic prediction (GBLUP-GP). We derived equations that describe the bias from low-coverage sequencing as an effect of binomial sampling of sequence reads, and allowed for any ploidy level of the sample considered. This allowed us to combine individual and pool genotypes in one GRM, treating pool-genotypes as a polyploid genotype, equal to the total ploidy-level of the parents of the pool. Using simulated data, we verified the magnitude of the GRM bias at different coverage depths for three different kinds of ryegrass breeding material: individual genotypes from single plants, pool-genotypes from F_2_ families, and pool-genotypes from synthetic varieties. To better handle missing data, we also tested imputation procedures, which are suited for analyzing allele-frequency genomic data. The relative advantages of the bias-correction and the imputation of missing data were evaluated using real data. We examined a large dataset, including single plants, F_2_ families, and synthetic varieties genotyped in three GBS assays, each with a different coverage depth, and evaluated them for heading date, crown rust resistance, and seed yield. Cross validations were used to test the accuracy using GBLUP approaches, demonstrating the feasibility of predicting among different breeding material. Bias-corrected GRMs proved to increase predictive accuracies when compared with standard approaches to construct GRMs. Among the imputation methods we tested, the random forest method yielded the highest predictive accuracy. The combinations of these two methods resulted in a meaningful increase of predictive ability (up to 0.09). The possibility of predicting across individuals and pools provides new opportunities for improving ryegrass breeding schemes.

## Introduction

Perennial ryegrass (*Lolium Perenne* L.) is the most valuable forage species in the temperate regions of northwest Europe, America, South Africa, Japan, Australia, and New Zealand (Humphreys et al., 2010). Traditionally, ryegrass breeding programs use recurrent selection based on genetic merit estimated from recorded phenotypes. This system results in a moderate genetic gain of about 7% per decade (Hayes et al., 2013). Efforts to reshape ryegrass breeding programs by introducing marker information, led to the conclusion that markers in candidate genes for complex traits generally explains only a small proportion of observed variance (Hayes et al., 2013), thus limiting the efficacy of marker-assisted selection (MAS) approaches (Jannink et al., 2010).

Genomic prediction (GP) (Meuwissen et al., 2001) allows one to perform selection based on genomic estimated breeding values (GEBVs) derived from dense genome-wide DNA markers. Recently, Fè et al. (2015, 2016) proposed GP as an effective way to use high-density marker information to overcome the limitations of MAS in ryegrass breeding. Fè et al. (2015, 2016) performed GBLUP-GP analysis on bi-parental family-pools and reported medium- to high-predictive ability (PA) for both disease resistance and quantitative agronomic traits. The bi-parental families were genotyped using a genotyping-by-sequencing (GBS) assay, which proved to be an efficient way to genotype ryegrass pools of heterogeneous individuals (Byrne et al., 2013). At each SNP locus of each sample, GBS provides a number of sequence-reads classified into two sets: sequences carrying the reference allele (S_R_) and sequences carrying the alternative allele (S_A_). The sum of S_R_ and S_A._ is the coverage depth or the total number of sequences (S_T_). The ratio between S_A_ and S_T_ gives an estimate of the true allele-frequency for the sample at the SNP locus. Such a frequency can be used as an SNP score, and for family pools, it should be interpreted as an estimate of the proportion of alternative alleles across all the individuals within the pool.

One premise of using GP in breeding programs is that samples can be genotyped at a lower cost than phenotyping them (Meuwissen et al., 2001; Goddard and Hayes, 2007). Increasing sample multiplexing is a straightforward way to reduce sequencing costs, but it results in a reduction of coverage depth (S_T_) (Elshire et al., 2011). However, reducing S_T_ in ryegrass family pools leads to a decline in the accuracy of the allele-frequency estimate (Ashraf et al., 2016). In addition, (Ashraf et al., 2016) showed that genomic relationship matrices (GRM) calculated by using low-S_T_ SNPs, are biased toward higher diagonal values, resulting in underestimates in genomic heritability. This bias in diagonal values is a consequence of over-estimating inbreeding and underestimating heterozygosity at low S_T_. A method to correct this bias is needed in order to utilize low-S_T_ genomic data for GBLUP-GP.

Reducing coverage depth also increases the fraction of missing data. This has been reported to be one of the main problems working with GBS data (Beissinger et al., 2013). Imputation of missing genotypes has been shown to be an effective approach for both increasing power in association studies (Marchini and Howie, 2010) and mitigating losses in accuracy in GP (Poland et al., 2012; Rutkoski et al., 2013). Several highly-accurate methods have been developed to assign allelic states to missing values in genotype data (reviewed by Marchini and Howie, 2010). However, these methods require using a high-quality reference genome (with chromosome-scale pseudomolecules), which is still not available for ryegrass. Efficient, haplotype-independent imputation methods exist, such as those implemented in Linkimpute (Money et al., 2015, 2017); however, such methods were developed for standard marker coding as counts of alleles, and so cannot be applied to pool allele-frequencies. The unavailability of a high-quality, reference genome for ryegrass (with chromosome-scale pseudomolecules) and our need to code markers as allele-frequencies, means that we must find alternative, haplotype-independent, imputation strategies for use in ryegrass.

Although GP has reportedly succeeded in ryegrass, additional studies are needed to determine how to efficiently use GP in breeding programs. Ryegrass breeders typically follow the following steps to develop new varieties: (1) parental individuals, selected from elite varieties, are crossed to generate F_1_ progenies, (2) seeds from each F_1_ are multiplied in isolation to generate F_2_ families that are then phenotyped in several replicates as family pools, (3) single plants (SPs) from selected F_2_ families are evaluated as individual genotypes, (4) synthetic varieties (SYNs) are constructed by polycrossing several SPs from the best performing F_2_ families (generally between 6 and 10 parents), (5) SYNs are maintained and evaluated as family pools, and after selection, (7) the best-performing SYNs are submitted for official testing (Detailed reviews of breeding methods for grasses are presented by Vogel and Pedersen, 1993; Hayes et al., 2013). In the present work, we considered data for all three kinds of breeding material (SPs, F2-families, and SYNs). Careful considerations on steps to be improved by GP are still needed. One important contribution would be to develop procedures that are capable of predicting the performances of individuals from the pools. In particular, because certain phenotypes cannot be measured in individuals, it would be useful to predict individual SP from pool-data on F2-families and/or SYNs. This would increase the efficiency of selecting new SYN parents. Therefore, the objectives of this study were to:

1. Derive a method for calculating GRM, using allele-frequencies for various kinds of family-pools, and for quantifying the factors that bias GRM diagonal elements (including low S_T_).
2. Derive a method to correct for biased GRM diagonal elements (due to low S_T_)_._
3. Compare haplotype-independent methods for imputing missing genotypes scored as continuous allele-frequencies.
4. Test the effectiveness of imputation strategies and bias correction on predictive ability within and across different breeding materials.

## Materials and methods

### Genomic data simulation and expected GRM

Genomic data were simulated for every ryegrass breeding material considered: single plants (SPs), F_1_ and F_2_ families, and synthetic varieties (SYNs). The simulated genomic data were used to calculate genomic relationship matrices (GRM). Various genomic data were produced to: (1) define the magnitude of unbiased GRM diagonal elements for individuals and various types of pools, (2) quantify the effects of factors influencing the GRM diagonal elements (such as small population size, number of contributing parents, inbreeding within the family-pools, and low S_T_), and (3) test a method to correct for biases due to low S_T_. To do this, genomic data were simulated as follows:

1. We generated 5,000 independent SNP markers for 300 parent pairs (parents were assumed to be unrelated) with an allele-frequency (*p*) sampled from a β-distribution with parameters α = 2 and β = 8.
2. We created 300 F_1_ family-pools by simulating crosses between each parent pair. Various F_1_ family-pool sizes were tested, ranging from 5 to 100 individuals.
3. We generated 300 F_2_ families, each created by simulating crossings between pairs of randomly selected F_1_ individuals (within the same F_1_ family pool). Each of the F_2_ individuals created by crossing two F_1_ plants was considered to be a single plant (SP). Pools of all the SPs originating from the same F_1_ family crosses were considered to be members of the same F_2_ family. Various F_2_ family population sizes were tested, ranging from 5 to 100 individuals. The genotypes of SPs belonging to the same F_2_ family were then averaged to generate F_2_-family allele frequencies.
4. We generated 300 SYNs, each generated by crossing 8 SPs randomly selected from different F_2_ families. (None of the crossed SPs were selected from the same F2 family.) We used 8 SPs because this was the average number of parents used to generate the SYNs in our real data (introduced later). The number of individuals generated by each cross was set to 50. This is because the results of F_1_ and F_2_ families showed that 50 individuals were enough to avoid allele drift due to small population size. Then the genotypes of individuals belonging to the same SYN were averaged to generate SYN allele frequencies.

Following this simulation procedure, true allele frequencies (*p*) were created for F_2_ families, SPs and SYNs. Allele-frequency data (ranging between zero and 1.0) were continuous for pools of F_2_ families and SYNs (resulting from average genotypes of several individuals), while data were discrete for individual SPs (equal to either zero or 1.0 for the two contrasting homozygous genotypes, and 0.5 for the heterozygous genotype).

For each breeding material, the simulated, true allele frequencies (*p*) were used to produce estimated allele frequencies ($\hat{p}$) for S_T_ values ranging from 1 to 100. This was done by random sampling S_T_ reads, wherein P(S_A_) = *p* and P(S_R_) = *1–p*. Estimated allele-frequencies were calculated as $\hat{p}$ = S_A_/S_T_. (Missing values were not considered in the simulation.) GRM were computed using true allele frequencies and estimated allele frequencies at different S_T_ values. We also computed GRM corrected for low S_T_ inaccuracies, using the method described later.

### Plant material and phenotyping

The phenotypic data we used were derived from a standard diploid ryegrass breeding program conducted at DLF Seeds A/S (Store Heddinge, Denmark). Three different kinds of breeding material, commonly produced in ryegrass breeding programs, were present:

1. SPs: 1,225 single plants, produced in 2014 from 50 different F_2_ families.
2. F_2_ families: 1,791 bi-parental F_2_ families, phenotyped and genotyped as pools, produced between 2000 and 2012.
3. SYNs: 127 multi-parental, synthetic families obtained by crossing from 6 to 10 randomly-selected single plants from superior F_2_ families, phenotyped and genotyped as pools.

For F_2_ families and SYNs, phenotypic measurements were based on replicated sward plots for each family, for which only family means were recorded. For SPs, individual phenotypes were obtained. The following agronomic traits were considered:

1. Heading date (HD), defined as days after May 1, in which plants start showing at least one spikelet per tiller. HD is available for all breeding material.
2. Crown rust resistance (CRR), measured by visual scoring during the period of maximum infection. The scale ranged from 1 (plant completely covered by rust) to 9 (no rust symptoms). CRR is available for all breeding material.
3. Seed yield (SY), expressed in g m^−2^. This trait was scored only for F_2_ families and SYNs.

The phenotype data for F_2_ families and the SYNs were scored over several years across different locations. All fields were organized into trials that were further divided into plots. Detailed descriptions of phenotyping strategy and field design are given in Fè et al. (2015, 2016).

SP fields were organized by sowing groups of 50 SPs collected from the same F_2_ family in separate rows (There were no replicates of the genotype). The score for CRR was collected in 2014 in Les Alleuds (France). The SPs were sown during spring and the CRR was scored in September after a natural crown rust attack. Heading date (ear emergence date) was assessed in 2014 in Store Heddinge, Denmark. SPs were sown during late summer and scored during the following season.

### Plant genotyping

Sequence data were produced using GBS approach, with the methylation-sensitive restriction enzyme ApeKI to target the low copy fraction of the genome. Sampling and library preparation followed the protocol described by Byrne et al. (2013). F_2_ families and SYNs were genotyped based on a pooled sample from the family.

Plant materials were genotyped in three rounds by using an Illumina HiSeq2000 (100 bp single-end) genome sequencer. Different multiplexing set-ups were used in these three assays:

Assay 1: consisting of 16 libraries containing maximum 64 samples per library. Each library was sequenced using four lanes (995 F_2_ families were included in this assay).

Assay 2: consisting of 14 libraries containing maximum 96 samples per library. Each library was sequenced using four lanes (all 1,225 SPs and 39 SYNs were included in this assay).

Assay 3: consisting of 16 libraries containing maximum 64 samples per library. Each library was sequenced using four lanes (796 F_2_ families and 89 SYNs were included in this assay).

The sequencing data from the three assays were aligned against a draft sequence assembly to produce common SNP calls for all the samples (*sensu* Byrne et al., 2015). Markers with a missing rate above 0.5, and a MAF lower than 0.01 were excluded. A total of 897,426 SNP frequencies distributed across 26,384 scaffolds were available for further analyses.

### Imputation methods

Three methods were used to impute missing SNP data: mean imputation (MNi), k nearest neighbor imputation (kNNi), and random forest imputation (RFi). Imputation was carried out scaffold by scaffold for kNNi and RFi. For MNi, each missing data point *x*_*ij*_ for pool *i* marker *j* was replaced with the mean ${\bar{x}}_{ij}$ of the non-missing values for marker *j* of other individuals or pools. For kNNi, each missing data point was imputed by replacing it with the weighted average of the data points at the *k* closest markers (Troyanskaya et al., 2001). Specifically, for each marker *j*, all other markers were first sorted according to the Euclidean distance to marker *j*. Each marker was included twice, both in the original and flipped state (1 minus the pool or individual allele frequency), to ensure that markers in strong negative linkage disequilibrium were also considered to impute the marker under analysis. Subsequently, for each row *i* of marker *j*, the weighted average of the *k* closest markers at row *i* were used to estimating the marker value at data point *x*_*ij*_. The weight of each marker was assigned 1/*d*^2^ where *d* was the Euclidean distance between marker *j* and the marker to be weighted. The *k* parameter was set to *k* = 6 after testing the accuracy of the imputation for each data set.

For RFi, missing marker values were estimated using a random forest regression algorithm (Breiman, 2001) as implemented in the R package “random Forest” (Liaw and Wiener, 2002). Random forest is a machine-learning algorithm that uses a group of decision trees to determine a classification or to predict a value for a new instance. RFi starts by first imputing all missing marker values using MNi. Subsequently, the algorithm estimates and updates missing markers as follows: (1) for the first marker *j*, a group average of 100 regression trees were grown (for each regression tree, the algorithm generated a bootstrap sample of non-missing individuals and a random sample of markers), (2) missing values for marker *j* were predicted as group averages of the 100 trees applied to the other markers, (3) the imputed marker *j* was updated on the marker matrix, (4) steps one to three were repeated for all the markers, and (5) steps one to four were repeated with new imputed markers, for a maximum of 10 iterations or until the difference between the newly-imputed and the last-imputed dataset began to diverge.

The imputation accuracy for MNi and RFi was estimated by masking 0.1% of observed values for each marker with missing values. After imputing these data points, the accuracy was described using R^2^ defined as:

(1) $$\begin{array}{l} R^{2}=1-\frac{\sum_{j}{\left(x_{j}true-\;x_{j}\;imputed\right)}^{2}}{\sum_{j}{\left(x_{j}true-\;mean\left(x\right)\right)}^{2}} \end{array}$$

where *j* was iterated across all the masked values. Ten replicates of this simulation were carried out on 10% of randomly-selected scaffolds.

### GRM calculation and bias correction

GRM calculations were based on VanRaden (2008), adapted to use allele frequencies (ranging between 0 and 1) rather than allele-variants. First, allele frequencies were arranged in a matrix **F**_ij_, with *i* indexing the samples and *j* indexing the markers. The matrix was then centered by the mean SNP frequencies (**M**_*j*_ = **F**_*j*_ – ${\bar{\text{F}}}_{j}$). When working with allele-frequencies, the mean of all allele-frequency samples at a given SNP is equivalent to the minimum allele frequency (MAF or$\hat{\;p}$). **M** was then used to compute **G**, as follows:

(2) $$\begin{array}{l} \text{G}={\text{MM}}^{\prime }/\sigma_{G}^{2} \end{array}$$

where $\sigma_{G}^{2}$ is a scaling parameter, corresponding to the sum of the expected SNP variance across genotypes, as computed by Ashraf et al. (2014):

(3) $$\begin{array}{l} \sigma_{G}^{2}=\frac{1}{n}\sum_{j=i}^{m}{\hat{p}}_{j}\left(1-\;{\hat{p}}_{j}\right) \end{array}$$

where *m* equals the number of markers, ${\hat{p}}_{j}$ equals the frequency of the *jth* marker, and *n* represents the ploidy number of the breeding material under analysis. The average genotype of a family pool can be considered to be polyploidal genotype with a ploidy level equal to the sum of the ploidy number of the parents used to generate it (a conceptual demonstration of this assumption is shown for F_2_ families by Ashraf et al., 2014). Ploidy levels of 2, 4, and 16 were considered for SPs, F_2_ families, and SYNs, respectively. Using this scaling factor, the expected diagonal element of **G** is equal to one plus the inbreeding coefficient. When the GRM includes different breeding materials, we used *n* = 4, so that diagonal elements were scaled relative to the F_2_ families.

Because SNP frequencies are estimated by sequencing a finite number of reads (S_T_), they are affected by binomial samplings that increase the variance of SNP frequencies ($\sigma_{G}^{2}$). This extra variance can be derived using a normal approximation for the binomial distribution, as described in the following equations. The number of alternative alleles observed for each pool at each SNP is distributed as (normal approximation):

(4) $$\begin{array}{l} {\text{S}}_{\text{A}}\sim \;N\left({{\text{S}}_{\text{T}}\;p}_{\;},\;{{\text{S}}_{\text{T}}\;p}_{\;}\left(1-p\right)\right) \end{array}$$

where S_T_ is the number of observed reads and *p* is the true marker frequency. The observed allele-frequency estimate ($\hat{p}$) from sequence reads is obtained by dividing S_A_ by S_T_. The sampling distribution of $\hat{p}$ at a specific locus in a pool with a true allele frequency *p* can be described as:

(5) $$\begin{array}{l} \hat{p}\;\sim \;N\left(p,p\left(1-p\right)/{\text{S}}_{\text{T}}\right) \end{array}$$

The binomial error variance for the genotype estimate is therefore *p*(1−*p*)/*S*_*T*_, while the expected binomial variance ($\sigma_{Bin}^{2}$) is:

(6) $$\begin{array}{l} \text{E}\left[p\left(1-p\right)S_{\text{T}}\right]=\frac{1}{{\text{S}}_{\text{T}}}\left(\text{E}\left[p\right]-\text{E}\left[p^{2}\right]\right)\\ =\frac{1}{{\text{S}}_{\text{T}}}\left(p-\frac{1}{\text{n}}p\left(1-p\right)-p^{2}\right)\\ =\frac{1}{{\text{S}}_{\text{T}}}\left(1-\frac{1}{\text{n}}\right)p\left(1-p\right) \end{array}$$

which converges to zero as S_T_ increases.

For instance, the binomial error variance for a diploid F_2_ family will be $\frac{3}{4}\frac{p\left(1-p\right)}{{\text{S}}_{\text{T}}}$. The same conclusion was reported by Ashraf et al. (2014) using a different derivation. Equation 6 generalizes the expression to any ploidy or any number of contributing parents.

When the GRM is computed, the diagonal element obtained represents the sum of squared allele-frequencies over the total number of SNPs (*m*). This diagonal is inflated because it includes the binomial variances of all the allele frequencies. The expected binomial variance from all *m* SNPs due to low sequencing depth (${\hat{\sigma }}_{Bin}^{2}$) of the family pool *i* is equal to:

(7) $$\begin{array}{l} {\hat{\sigma }}_{Bin_{i}}^{2}=\sum_{j=1}^{m}\left(1-\frac{1}{n_{i}}\right){\hat{p}}_{j}\left(1-{\hat{p}}_{j}\right)/{S_{\text{T}}}_{ij}\\ =\left(1-\frac{1}{n_{i}}\right)\;\sum_{j=1}^{m}{\hat{p}}_{j}\left(1-{\hat{p}}_{j}\right)/S_{{\text{T}}_{ij}} \end{array}$$

where $\hat{p_{j}}$ is the observed allele frequency for SNP *j, n* is the assumed ploidy number of the pool, and _S_T_*ij*_ is sequencing depth for pool *i* and SNP *j*.

The observed marker variance ($\sigma_{G}^{2}$) of the pool *i* is equals to:

(8) $$\begin{array}{l} {\hat{\sigma }}_{G_{i}}^{2}=\frac{1}{n_{i}}\sum_{j=1}^{m}{\hat{p}}_{j}\left(1-{\hat{p}}_{j}\right) \end{array}$$

This variance is inflated due to binomial variance. The inflation (ω) can be defined for each sample as the fraction of the total marker variance that is due to binomial sampling, and is equal to:

(9) $$\begin{array}{l} \omega_{i}=\frac{{\hat{\sigma }}_{Bin_{i}}^{2}}{\left({\hat{\sigma }}_{Bin_{i}}^{2}+{\hat{\sigma }}_{G_{i}}^{2}\right)}\;\\ =\frac{\left(1-\frac{1}{n_{i}}\right)\;\sum_{j=1}^{m}{\hat{p}}_{j}\left(1-{\hat{p}}_{j}\right)/{{\text{S}}_{\text{T}}}_{ij}}{\frac{1}{n_{i}}\sum_{j=1}^{m}p_{j}\left(1-p_{j}\right)+\;\left(1-\frac{1}{n_{i}}\right)\;\sum_{j=1}^{m}{\hat{p}}_{j}\left(1-{\hat{p}}_{j}\right)/{{\text{S}}_{\text{T}}}_{ij}}\\ =\frac{n_{i}-1}{{{\bar{{\text{S}}_{\text{T}}}}_{i}+\;n}_{i}-1} \end{array}$$

which is derived by substituting ${\hat{\sigma }}_{Bin_{i}}^{2}$ of Equation 7 and ${\hat{\sigma }}_{G_{i}}^{2}$ of Equation 8, and by defining an average S_T_ (${\bar{\text{S}}}_{\text{T}}$) for each individual across all SNPs. Equation 9 shows that the inflation in genomic variance (due to binomial sampling) does not depend on allele frequency, rather it only depends on the ploidy number and the average S_T_ (coverage depth) of the sample. Corrected GRM values were calculated by scaling down the diagonal elements of each individual according to ω_*i*_ as follows:

(10) $$\begin{array}{l} Dc_{i}=Db_{i}\left(1-\omega_{i}\right) \end{array}$$

where *Db*_*i*_ is the *ith* element of the biased diagonal element in **G**, while *Dc*_*i*_ is the corrected element.

### Statistical models and cross-validation schemes

The phenotypic data were analyzed using linear mixed models. Genomic information was incorporated by the Genomic Best Linear Unbiased Prediction (GBLUP) method (Habier et al., 2007; VanRaden, 2008). We adopted the following model:

(11) $$\begin{array}{l} y=1\mu +\text{Xt}+Z_{1}\text{i}+Z_{2}\text{l}+e \end{array}$$

where **y** is a vector with phenotypic observations, μ is the overall mean, **1** is a vector of ones, **X** is the design matrix of fixed effects, and **t** is the vector of trial effects nested within location and year, **Z**_1_ and **Z**_2_ represent design matrices of random factors, **i** is a vector of genomic breeding values where **i** ~ N(0, **G**$\sigma_{i}^{2}$) where **G** is the genomic relationship matrix, **l** is a vector of interaction effects of genotype by location by year where **l** ~ N(0, **I**$\sigma_{ily}^{2}$), and ***e*** is a vector of random residuals where **e** ~ N(0, **I**$\sigma_{e}^{2}$).

Variance components were estimated using the restricted maximum likelihood method, using the software package DMU (Jensen et al., 1997; Madsen and Jensen, 2013). We compared models using GRMs calculated with genotype datasets imputed using three methods (MNi, kNNi, and RFi) with or without correction for diagonal bias. First, the phenotypes were corrected for fixed effects by running each model on the full dataset. Then, genomic estimated breeding values (GEBVs) were determined by masking phenotypes by using two different cross-validation procedures: a leave-one-out and an across-set cross-validation. In the leave-one-out procedure, one sample per iteration was excluded from the model training data and then the GEBV of the missing data point was predicted using the data from all other samples. In the across-set validation procedure, the genotypes belonging to each of the three different breeding materials (SPs, F_2_ families, and SYNs) were left out and GEBV of these data points then were predicted using the other two sets. The predictive abilities (PA) were computed as the Pearson's correlation coefficient between the phenotype corrected for the fixed effects (averaged across replicates for each sample) and the predicted GEBVs.

## Results

### Analysis of simulated data

SNP datasets were simulated to study the diagonal elements of genomic relationship matrices (GRM) for the three different ryegrass breeding materials (individuals and two types of pools). The GRM diagonal elements reflected the variance of family pools and were affected by at least four factors: (1) genetic drift due to small population size in the pools, (2) the number of contributing parents of the family pool, (3) the extent of inbreeding created in the F_1_ multiplication of the family pool, and (4) inaccuracies in the allele-frequency estimates due to low S_T_ values for the genomic data.

**Figure 1:**
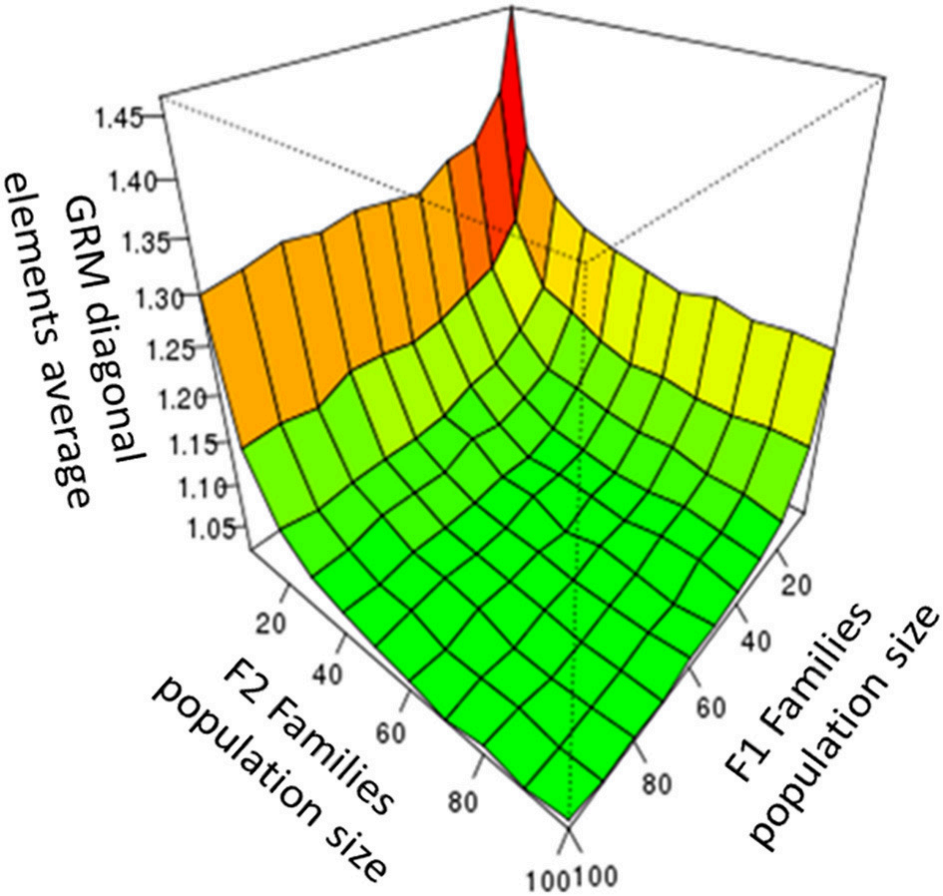
The average GRM diagonal elements observed for F_2_ families, as a function of the population size of the F_1_ and the F_2_ families.

We simulated different F_1_ and F_2_ family population sizes to investigate genetic drift effects when small population sizes are used (due to deviation from the Hardy-Weinberg (HW) equilibrium). GRM data were computed using true allele frequencies. An increase in GRM diagonal elements due to small population size was detectable (Figure 1). However, we found that around 50 individuals per family were sufficient to maintain the HW-equilibrium. Because breeding populations are typically much larger than 50 individuals, the effect of small population size was not further investigated and all simulations were produced using a population size of 50 individuals per family.

**Figure 2:**
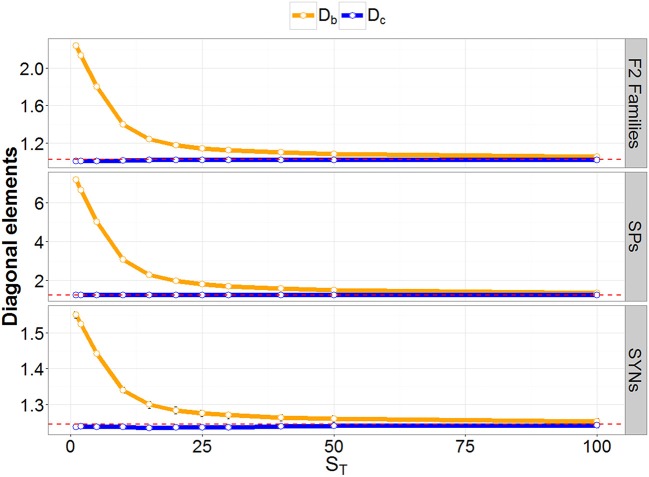
Result of the simulation study. Diagonal elements of GRM at different coverage depths (S_T_) are denoted before (Db: orange) and after (Dc: blue) correcting for low S_T_ bias. The red-dashed lines represent the diagonal element of the GRM calculated with true allele frequencies.

GRM values were also computed using simulated, true allele frequencies to investigate differences in the diagonal elements for different breeding materials. The average GRM diagonal for the single plants (SPs, coming from the F_2_ families) was equal to 1.25 (Figure 2), reflecting an inbreeding coefficient of 0.25. This result can be explained by the fact that SPs in F_2_ are generated by crossing F_1_ full sibs with an average co-ancestry of 0.25. However, the average GRM diagonals for the F_2_ families and for the SYNs were equal to 1.0, reflecting that there is no inbreeding effect on the mean family genotypes, i.e., the variance of means between the F_2_ families and the SYNs is not affected by the inbreeding within the families and SYNs. A theoretical derivation for the SPs and F_2_ family variances is given in Appendix I, based on standard quantitative genetic theory. This theoretically verifies the results for SPs and pools with two parents, accounting for both inbreeding and genetic drift.

GRMs were also calculated using observed allele frequencies at each S_T_. The resulting averages of the diagonal elements are reported in Figure 2. A large inflation of the GRM diagonal was observed for a low S_T_. Moderate inflation was still observed at a rather high S_T_, and the difference between the expected and observed inbreeding coefficients remained substantial until S_T_ was about 50. Diagonal elements of the GRM (calculated using observed allele-frequencies) were also corrected for binomial sampling error due to low S_T_, as described in Equation 9. The average corrected diagonal elements are displayed in Figure 2. They showed no inflation at all the S_T_ values we considered.

### Real data analysis

For practical application, we considered the three breeding materials, SPs (individuals), F_2_ families (pools), and SYNs (pools), genotyped in three different assays (using different multiplexing sequencing parameters) to investigate the relevance of bias correction and missing data imputation on the GRM values.

The three assays exhibited different S_T_ scores and different missing data fractions. Specifically, for Assay 1 S_T_ was 12.6 and its missing fraction was 20.4%; for Assay 2, S_T_ was 3.3 and its missing fraction was 58.5%, while for Assay 3, S_T_ was 13.4 and its missing fraction was 9.7%.

#### GRM bias correction and missing genotype imputation

**Figure 3:**
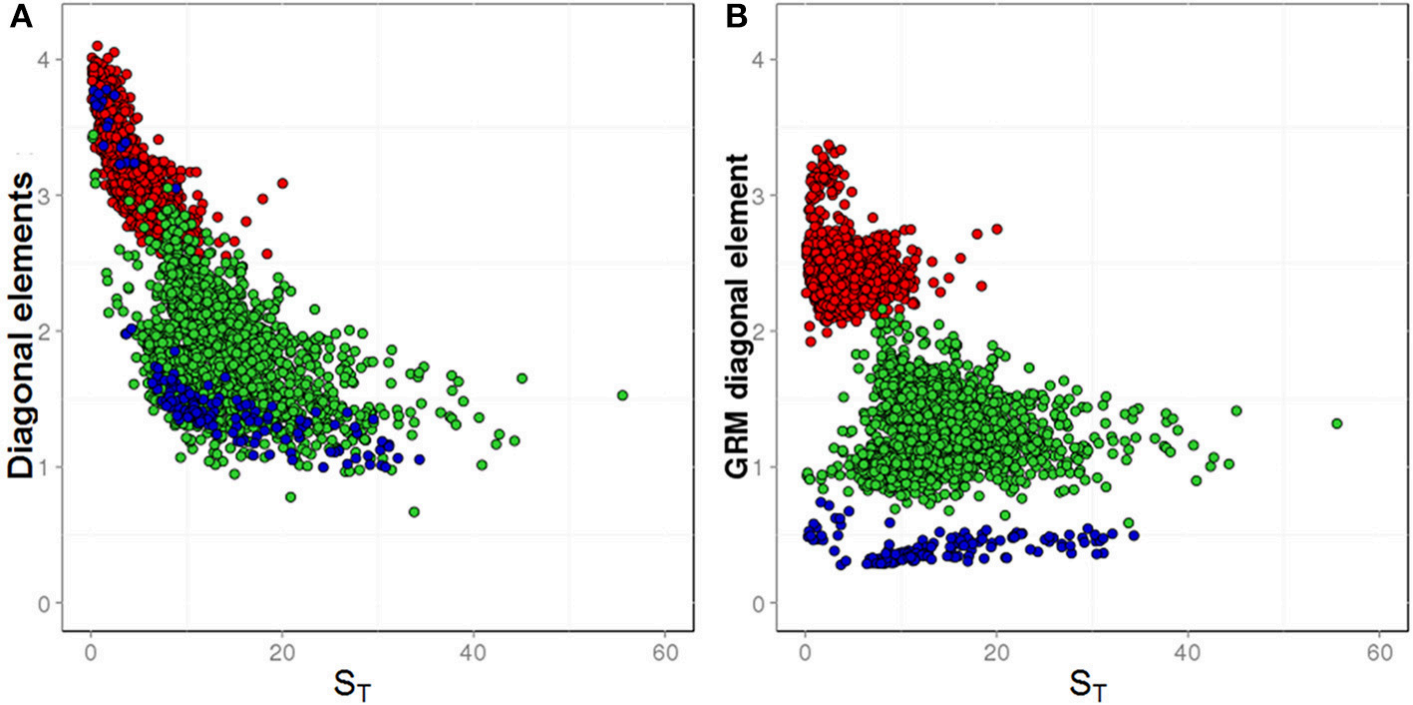
The diagonal elements of GRMs plotted against sample averages for coverage depths (S_T_). Different breeding materials are colored as follow: biparental F_2_ families (green dots), multiparental synthetic varieties (blue dots), and single plants (red dots). **(A)** Shows GRM diagonal elements before low S_T_-bias correction; **(B)** shows GRM diagonal elements after low S_T_-bias correction.

In Figure 3, the diagonal elements of GRMs are displayed as a function of the average sample S_T_ across all markers. This figure shows diagonal elements before (Figure 3A) and after (Figure 3B) the bias correction was performed. As described above, based on simulated data and use of pool frequencies without error, the expected average diagonal value for F_2_ families is 1.0. In the real data, the average diagonal value for F_2_ families observed before correction was 1.81, and the diagonal elements were not randomly distributed around the mean, showing a correlation with the sample S_T_ (Figure 3A, green dots). After correction, the average diagonal value decreased to 1.35 and the diagonal elements were randomly distributed around the mean (Figure 3B, green dots).

The expected average diagonal value of an SP would be 1.25, if scaled according to its own ploidy level. However, in our study, the GRMs were scaled to the ploidy level of F_2_ families, which is twice the ploidy level of the SPs. With this different scaling, the expected average diagonal value of SPs would be 2.5. In the real data, the observed average diagonal value of SPs before bias correction was 3.17, while it declined to 2.47 after the bias correction.

The expected average diagonal value of an SYN would be 1.0, if scaled according to its own ploidy level. However, the GRMs in our study were scaled to the ploidy level of F_2_ families, while SYNs were pools of genotypes derived from a polycross of 6 to 10 SPs. Therefore, SYNs have an assumed ploidy between 3-and-5 times higher than the F_2_ families and the expected average diagonal value for SYNs with this different scaling should be between 0.33 and 0.20. The observed average diagonal elements of the GRM were divided in two groups, the first (SYNs genotyped in Assay 2 with low S_T_ scores), showed an average value of 3.46, while the second group (SYNs genotyped in Assay 3 with high S_T_ scores) showed an average value of 1.38 (Figure 3A, blue dots). After correction for depth bias, all the diagonal elements of SYNs clustered together with an average value of 0.38 (Figure 3B, blue dots).

Accuracies associated with imputing missing SNPs were calculated as R^2^ between the observed and imputed values and were: 0.5, 0.73, and 0.77 for MNi, kNNi, and RFi, respectively. The computation time required for calculating kNNi was relatively short (2.7 h for the full genomic dataset) on a standard computer, whereas the RFi computation required about 110 times more processing time.

#### Accuracies of genomic predictions

**Figure 4:**
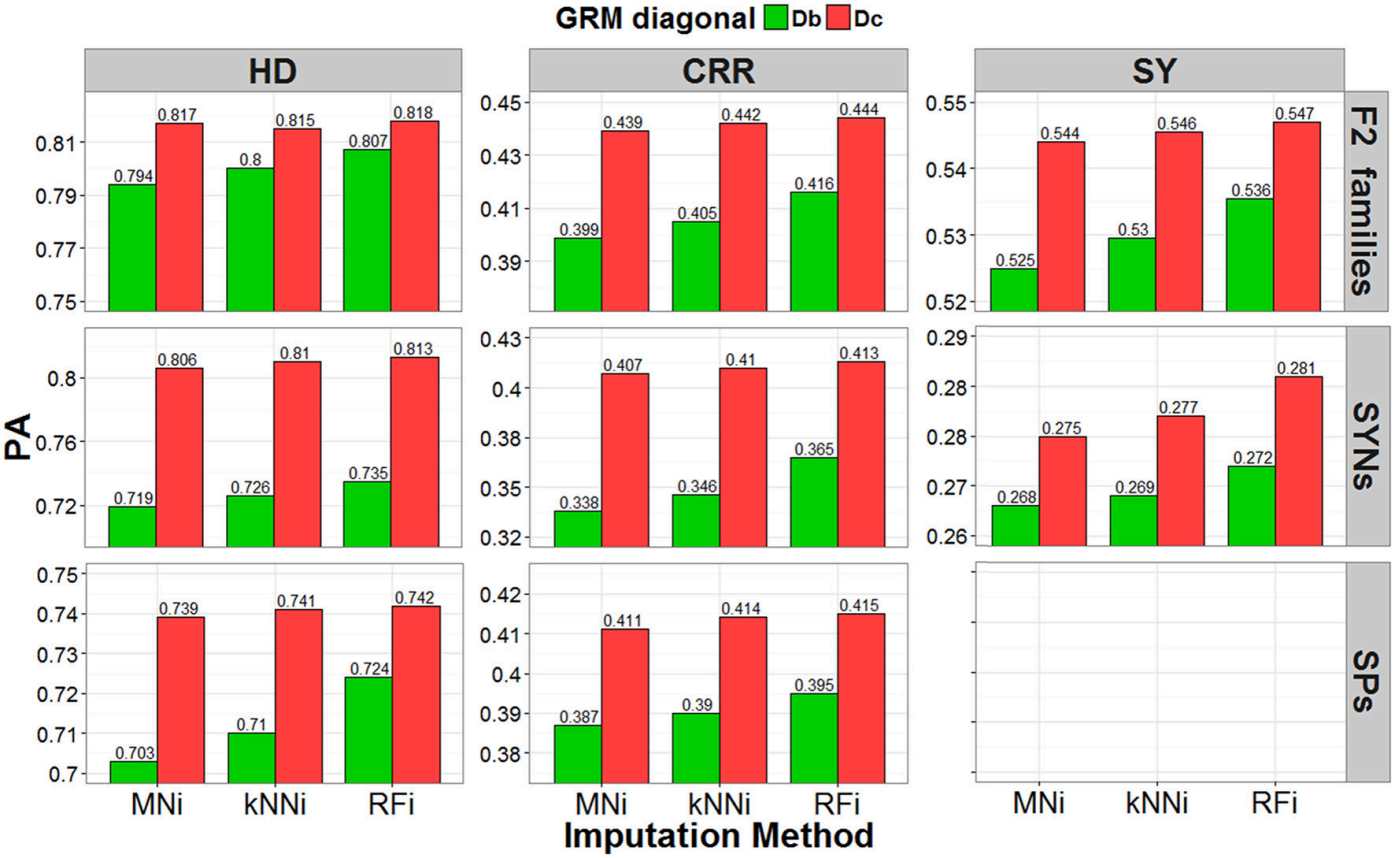
Predictive ability (PA) estimated with the leave-one-out cross-validation strategy. Result obtained by using three different imputation strategies (mean imputation MNi, k-nearest-neighbor kNNi, and random forest RFi) and two bias correction procedures for the allele-frequencies estimates (biased diagonal Db and corrected diagonal Dc), for three different traits (heading date HD, crown rust resistance CRR, and seed yield SY) in F2 families (pools), SYNthetic varieties (pools) and Single Plants.

**Figure 5:**
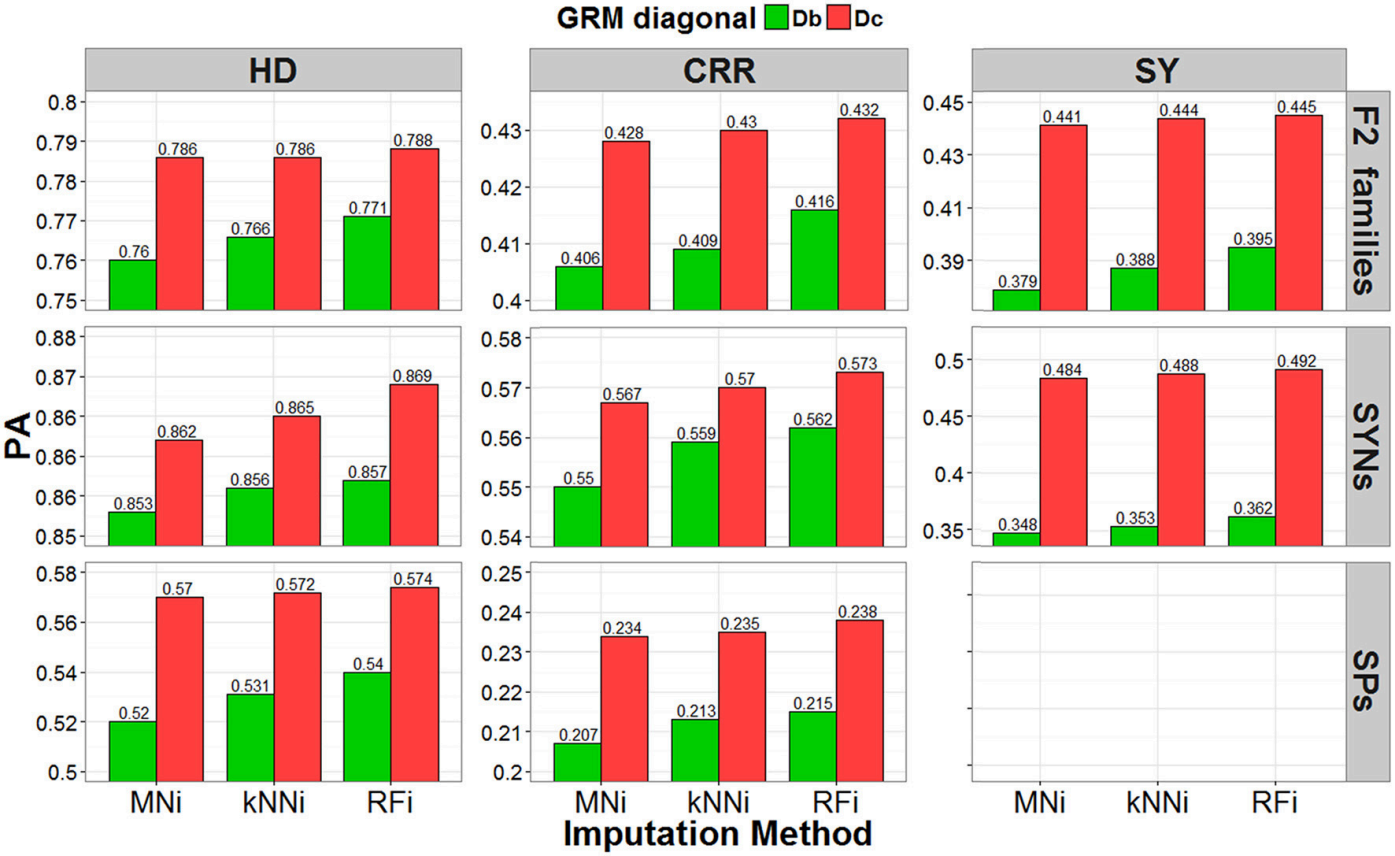
The predictive ability (PA) estimated with the across-set cross-validation procedure. Result obtained by using three different imputation strategies (mean imputation MNi, k-nearest-neighbor kNNi, and random forest RFi) and two bias-correction procedures for the allele-frequencies estimates (biased diagonal Db and corrected diagonal Dc), for three different traits (heading date HD, crown rust resistance CRR, and seed yield SY) in F2 families (pools), SYNthetic varieties (pools) and Single Plants.

Predictive abilities (PAs) of GBLUP-GP were evaluated by cross validation using GRMs calculated using SNP data imputed with each of the three imputation methods (MNi, kNNi and RFi), and with and without correction for low S_T_ scores. Results are presented for the leave-one-out cross validation strategy within each breeding material (Figure 4) and for the across-set, cross-validation procedure (Figure 5). Using GRM corrected for low S_T_ bias yielded higher PAs for each of the three breeding materials and for each of the three traits, regardless of the cross-validation strategy used. The RFi imputation was the method that led to the most accurate estimates for breeding values, followed by the kNNi and the MNi imputation methods. The highest PAs were observed when the RFi-imputed data were used together with a correction for low S_T_ scores. We observed larger PAs in scenarios that used MNi imputations than in scenarios where no corrections were made.

When using the leave-one-out cross-validation procedure, the largest PA (predictive ability, correlation of GEBV with corrected phenotype) was for predicting crown rust resistance in F_2_ families (PA range: 0.399–0.444), for predicting heading date for synthetic varieties (PA range: 0.719–0.813), and for predicting heading date for single plants (PA range: 0.703–0.742). In the across-set cross-validation procedure, the largest PA was for seed yield for F_2_ families (PA range: 0.379–0.445), seed yield for synthetic varieties (PA range: 0.348–0.492), and heading date for single plants (PA range: 0.52–0.574).

The leave-one-out procedure yielded the highest PAs for single plants and F_2_ families, while the across-set cross-validation procedure yielded the highest PAs for synthetic varieties. Finally, we observed that the correction for low S_T_ bias had a larger positive effect on PA than the effect of imputation strategy.

## Discussion

In our study, simulations were performed to depict the expected inbreeding of various ryegrass breeding materials (individual single plants, biparental F_2_ families and multiparental synthetic varieties as pools) and to quantify bias introduced in estimating inbreeding when low sequence depth (S_T_) GBS assays were used. We also derived methods for correcting for bias in genomic relationship matrices (GRM) that were calculated using genomic data at low S_T._ These simulations yielded unbiased estimates of the relative inbreeding level for different breeding materials.

Phenotypic and genotypic data for the different breeding materials were obtained from a commercial breeding program and were used to show the effectiveness of the proposed bias correction method to increase the predictive ability (PA) in cross validation. We showed that several ryegrass breeding materials could be combined into one unbiased GRM. Predictions of each breeding material were successfully carried out, based on information from the other types of breeding material.

### Correction for bias in GRM

Genotyping by sequencing (*sensu* Elshire et al., 2011) is a simple and robust genotyping approach that has been proposed to estimate allele frequencies in populations and family-pools (Byrne et al., 2013). The cost of plant phenotyping is relatively low compared to the costs of various genotyping approaches. Therefore, replacing some field evaluations with genotyping is only attractive when genotyping is also inexpensive. From this perspective, GBS is becoming advantageous because its cost per unit is low and it is continually declining. Furthermore, GBS can be made especially cost-effective when coverage depth is low (Barabaschi et al., 2015). However, if the depth of coverage is low, GBS will also produce a high amount of missing data (Beissinger et al., 2013). A low coverage depth (S_T_) also decreases the accuracy of allele-frequency estimates in the samples considered, which introduces biases that negatively affect heritability estimates, mapping, and genomic prediction (Ashraf et al., 2014).

Ashraf et al. (2014) showed that estimates of allele effects in association studies are biased downwards when allele frequencies are used that contain estimation errors due to low S_T_. Ashraf et al. (2016) were also the first to report that diagonal elements of GRMs are inflated when genotyping SNPs with low S_T_. This finding was confirmed in our study and can be explained by inflated diagonals in the GRMs that falsely indicate a high amount of inbreeding in the analyzed samples. Moreover, the simulations presented in our work showed that bias was high at low S_T_, but still detectable at medium-to-high coverage depths (~50).

Measuring error at low S_T_ represents a limitation to the routine use of genetic markers in ryegrass breeding schemes because: (1) low-S_T_ assays are often planned to minimize the costs, (2) different breeding materials, which are differently affected by the magnitude of sequencing errors, will often have to be included in the same genomic prediction analysis, and (3) new rounds of genotyping assays, which may differ in their coverage depth, will have to be used every year. One important outcome of our work is that we were able to develop a method that can efficiently remove bias due to measuring errors in allele frequencies (even at very low coverage depths) and can be extended to all the different breeding materials (individuals and various pools) used in the ryegrass breeding pipeline.

Increasing multiplex sequencing can be a successful strategy for reducing the cost or for increasing the number of genotyped samples that will enter the training population. Several studies have shown that the size of the training population is more important for predictive abilities, than the bias and missing data due to low coverage (Bassi et al., 2015). Finding the balance between cost and the size of the training set will be one of the principal challenges hindering future implementation of genomic prediction in ryegrass breeding programs.

We found that PA increased when bias-corrected GRMs were used. Also, in almost all cases, the bias-correction in the GRM provided a greater improvement in PA, than the improvements obtained from replacing the simple mean imputation with more advanced methods for imputation of missing genotype data. This indicates that bias due to low depth of coverage was more important than the loss of information resulting from an increased amount of missing data associated with a low depth of coverage.

### Imputation of missing genotype data

In the procedure of VanRaden (2008) for building GRMs, missing genotype data is replaced with the mean value of the non-missing genotypes, which we called mean imputation (MNi) method. Other, more advanced, imputation methods can be applied, but because our data consisted of allele frequency estimates on pools of individuals, and because there is no high-quality reference genome for ryegrass, we could only consider haplotype- and map-independent methods. We compared the k-nearest-neighbor (kNNi) and the random-forest (RFi) imputation methods as alternatives for MNi, and we found that there were two main advantages in using the kNNi and RFi methods: they were both map-independent and they could be relatively accurate.

The main factors that affect imputation accuracy of RFi and kNNi are the minor allele frequency (MAF) of the markers, the degree of relatedness between samples, and the linkage disequilibrium (LD) between markers (Rutkoski et al., 2013). Several studies have shown that SNP datasets with low MAF are easier to impute. This is because, at low MAF, missing markers can be quite accurately inferred just by using the most-frequent allele in the dataset (Hickey et al., 2012; Rutkoski et al., 2013). Moreover, the presence of closely-related samples in the dataset allows one to impute a missing marker data point by using information from markers more related to it, thus increasing the accuracy of the imputation (Hickey et al., 2012; Rutkoski et al., 2013).

Our study was conducted on ryegrass breeding material that consisted of groups of related samples sharing one or two parental lines. Moreover, this material had already been subjected to several rounds of selection, which may have reduced the variance in marker frequencies, resulting in rather low MAFs. These two elements enabled us to obtain adequate imputation accuracies. The ryegrass LD has been shown to decay after a few hundred bp, no matter what breeding material is used (Fè et al., 2015). Short-ranging LD has been related to less accurate imputation performances in several studies (Hickey et al., 2012; Rutkoski et al., 2013). This is because there is a reduced chance of using highly-correlated markers to aid imputation of missing data-points. Despite the low LD in ryegrass, the high marker density in our panel ensured that at least a proportion of them were close enough to be highly correlated, permitting us to obtain high imputation accuracies.

Another important finding of our study was the improved PAs we obtained after using both RFi and kNNi imputation procedures, compared to the standard MNi imputation. Although the gain in PA due to the choice of imputation strategy was not as large as the one resulting from our bias correction, a gain was still observed in all the different scenarios we examined. This result should promote the routine use of one of these two imputation methods (over standard MNi) to increase PA, by only adding a limited computing cost.

### Genomic prediction

The PAs we obtained demonstrate that using the genomic prediction approach for ryegrass breeding is very promising. Similar genomic prediction performances were reported for F_2_ families by Fè et al. (2015, 2016). However, we showed that it was possible to predict the breeding values of F_2_ families, single plants, and synthetic varieties and still ensure medium to high PAs, both by using data from the same breeding material, as well as from using the other kinds of breeding material as training data.

The potential for obtaining genomic-estimated breeding values (GEBV) for both individuals and pools was a key finding of ours that should lead to improvements in ryegrass breeding programs. For instance, it will be possible to accurately select single plants and generate synthetic varieties based on GEBVs of single plants. Phenotypic scores of SPs are difficult to generate for some key traits (e.g., yield-related traits), which can only be measured in plots consisting of several plants (Vogel and Pedersen, 1993). A precise evaluation of SP performances for these traits would require cloning each SP and use test-cross procedures (Hayes et al., 2013). However, cloning and maintaining a SP for the time needed to complete the evaluation of the test crosses is often considered too costly and too time-consuming. Therefore, SPs are often randomly selected from highly-performing F_2_ families, assuming that the performance of the F_2_ family reflects the SP's performance. Using SP GEBVs will increase the accuracy of SP selection for these traits that cannot be directly measured on single plants. Additionally, using SP GEBVs could allow a reduction in the generation interval required in breeding scenarios, for instance, because the phenotypic evaluation of F_2_ families could be avoided.

Although the predictive abilities (PAs) of SPs was considerably lower than the PAs observed for pools (F_2_ families and SYNs), the PA for a SYN resulting from crossing selected SPs would be higher. This is because the predicted breeding value of a SYN would be the average of the predicted breeding values of the SP parents of the SYN, and as explained previously, this average is more accurate due the averaging of the Mendelian-sampling genetic components in the SPs' breeding values.

## Conclusions

The methods we reported in this paper allowed us to gain better insight into using GBS data genomic prediction in different ryegrass breeding material (individual single plants, pools of F_2_ families and synthetic varieties). A bias was proven to affect the genomic relationship matrices (GRM) when low-to-medium sequencing depth (S_T_) GBS data were used, and this was verified via simulation. There was a bias introduced that was related to an overestimate of the inbreeding coefficient, resulting in inflated diagonal elements of the GRM. We presented a method for correcting this bias in GRMs, which proved to work correctly in simulated data.

The same bias is observed by using real genotypic data for ryegrass. Correcting this bias and applying haplotype-independent imputation methods greatly increased the PA of the approach. We expect this result will allow breeders to use low S_T_ genomic data, which will reduce the genotyping cost per sample. This approach would also reduce the economic effort associated with the use of genomic prediction, and potentially increase its effectiveness by increasing the number of the genotypes that can be included in training data sets.

We provided a method for calculating a GRM that can accommodate various types of ryegrass breeding material, in particular to combine indivuals and pools. This GRM allowed us to accurately predict BV across data sets. This finding can potentially reshape the ryegrass breeding industry. In particular, it will allow breeders to accurately predict the breeding value of complex traits for single plant parental lines, without the need for phenotypic testing.

## Author contributions

JJ, LJ, and FC designed the study. LJ and FC developed the theoretical part. IL, SB, and TA were responsible for the genotyping. IL processed the sequencing data. DF, MP, and CJ were responsible for collecting phenotypic data. FC developed the simulation study, performed the data analyses and wrote the draft manuscript. FC, LJ, and JJ edited and revised the final manuscript.

### Conflict of interest statement

The authors declare that the research was conducted in the absence of any commercial or financial relationships that could be construed as a potential conflict of interest.

## References

[B1] Ashraf B. H. Byrne S. Fé D. Czaban A. Asp T. Pedersen M. G.. (2016). Estimating genomic heritabilities at the level of family-pool samples of perennial ryegrass using genotyping-by-sequencing. Theor. Appl. Genet. 129, 45–52. 10.1007/s00122-015-2607-9 26407618 PMC4703624

[B2] Ashraf B. H. Jensen J. Asp T. Janss L. L. (2014). Association studies using family pools of outcrossing crops based on allele-frequency estimates from DNA sequencing. Theor. Appl. Genet. 127, 1331–1341. 10.1007/s00122-014-2300-4 24668443 PMC4035547

[B3] Barabaschi D. Tondelli A. Desiderio F. Volante A. Vaccino P. Valè G.. (2015). Next generation breeding. Plant Sci. 242, 3–13. 10.1016/j.plantsci.2015.07.010 26566820

[B4] Bassi F. M. Bentley A. R. Charmet G. Ortiz R. Crossa J. (2015). Breeding schemes for the implementation of genomic selection in wheat *(Triticum* spp.). Plant Sci. 242, 23–36. 10.1016/j.plantsci.2015.08.021 26566822

[B5] Beissinger T. M. Hirsch C. N. Sekhon R. S. Foerster J. M. Johnson J. M. Muttoni G.. (2013). Marker density and read depth for genotyping populations using genotyping-by-sequencing. Genetics 193, 1073–1081. 10.1534/genetics.112.147710 23410831 PMC3606087

[B6] Breiman L. (2001). Random forests. Mach. Learn. 45, 5–32. 10.1023/A:1010933404324

[B7] Byrne S. Czaban A. Studer B. Panitz F. Bendixen C. Asp T. (2013). Genome wide allele frequency fingerprints (GWAFFs) of populations via genotyping by sequencing. PLoS ONE 8:e57438. 10.1371/journal.pone.0057438 23469194 PMC3587605

[B8] Byrne S. L. Nagy I. Pfeifer M. Armstead I. Swain S. Studer B.. (2015). A synteny-based draft genome sequence of the forage grass *Lolium perenne*. Plant J. 84, 816–826. 10.1111/tpj.13037 26408275

[B9] Elshire R. J. Glaubitz J. C. Sun Q. Poland J. A. Kawamoto K. Buckler E. S.. (2011). A robust, simple genotyping-by-sequencing (GBS) approach for high diversity species. PLoS ONE 6:e19379. 10.1371/journal.pone.0019379 21573248 PMC3087801

[B10] Fè D. Ashraf B. H. Pedersen M. G. Janss L. Byrne S. Roulund N.. (2016). Accuracy of genomic prediction in a commercial perennial ryegrass breeding program. Plant Genome 9, 1–22. 10.3835/plantgenome2015.11.0110 27902790

[B11] Fè D. Cericola F. Byrne S. Lenk I. Ashraf B. H. Pedersen M. G.. (2015). Genomic dissection and prediction of heading date in perennial ryegrass. BMC Genomics 16:921. 10.1186/s12864-015-2163-3 26559662 PMC4642674

[B12] Goddard M. E. Hayes B. J. (2007). Genomic selection. J. Anim. Breed. Genet. 124, 323–330. 10.1111/j.1439-0388.2007.00702.x 18076469

[B13] Habier D. Fernando R. L. Dekkers J. C. (2007). The impact of genetic relationship information on genome-assisted breeding values. Genetics 177, 2389–2397. 10.1534/genetics.107.081190 18073436 PMC2219482

[B14] Hayes B. J. Cogan N. O. I. Pembleton L. W. Goddard M. E. Wang J. Spangenberg G. C. (2013). Prospects for genomic selection in forage plant species. Plant Breed. 132, 133–143. 10.1111/pbr.12037

[B15] Hickey J. M. Crossa J. Babu R. de los Campos G. (2012). Factors affecting the accuracy of genotype imputation in populations from several maize breeding programs. Crop Sci. 52, 654–663. 10.2135/cropsci2011.07.0358

[B16] Humphreys M. O. Feuerstein U. Vandewalle M. (2010). Fodder crops and amenity grasses, in Fodder Crops and Amenity Grasses, eds Boller B. Posselt U. K. Veronesi F. (New York, NY: Springer), 211–260.

[B17] Jannink J. L. Lorenz A. J. Iwata H. (2010). Genomic selection in plant breeding: from theory to practice. Brief. Funct. Genomics 9, 166–177. 10.1093/bfgp/elq001 20156985

[B18] Jensen L. Mantysaari E. A. Madsen P. Thompson R. (1997). Residual maximum likelihood estimation of (co)variance components in multivariate mixed linear models using average information. J. Indian Soc. Agric. Stat. 49, 215–236.

[B19] Liaw A. Wiener M. (2002). Classification and Regression by randomForest. R News 2/3, 18–22. 14632445

[B20] Madsen P. Jensen J. (2013). A User's Guide to DMU. Available online at: http://dmu.agrsci.dk/DMU/Doc/Current/

[B21] Marchini J. Howie B. (2010). Genotype imputation for genome-wide association studies. Nat. Rev. Genet. 11, 499–511. 10.1038/nrg2796 20517342

[B22] Meuwissen T. H. Hayes B. J. Goddard M. E. (2001). Prediction of total genetic value using genome-wide dense marker maps. Genetics 157, 1819–29. 11290733 10.1093/genetics/157.4.1819 PMC1461589

[B23] Money D. Gardner K. Migicovsky Z. Schwaninger H. Zhong G. Y. Myles S. (2015). LinkImpute: fast and accurate genotype imputation for nonmodel organisms. G3(Bethesda) 5, 2383–2390. 10.1534/g3.115.021667 26377960 PMC4632058

[B24] Money D. Migicovsky Z. Gardner K. Myles S. (2017). LinkImputeR: user-guided genotype calling and imputation for non-model organisms. BMC Genomics 18:523. 10.1186/s12864-017-3873-5 28693460 PMC5504746

[B25] Poland J. Endelman J. Dawson J. Rutkoski J. Wu S. Y. Manes Y. (2012). Genomic selection in wheat breeding using genotyping-by-sequencing. Plant Genome 5, 103–113. 10.3835/plantgenome2012.06.0006

[B26] Rutkoski J. E. Poland J. Jannink J. L. Sorrells M. E. (2013). Imputation of unordered markers and the impact on genomic selection accuracy. G3 (Bethesda) 3, 427–439. 10.1534/g3.112.005363 23449944 PMC3583451

[B27] Troyanskaya O. Cantor M. Sherlock G. Brown P. Hastie T. Tibshirani R.. (2001). Missing value estimation methods for DNA microarrays. Bioinformatics 17, 520–525. 10.1093/bioinformatics/17.6.520 11395428

[B28] VanRaden P. M. (2008). Efficient methods to compute genomic predictions. J. Dairy Sci. 91, 4414–4423. 10.3168/jds.2007-0980 18946147

[B29] Vogel K. P. Pedersen J. F. (1993). Breeding systems for cross-pollinated perennial grasses. Plant Breed. Rev. 11, 251–274. 10.1002/9780470650035.ch7

