# Supplementary material for: Optimized Use of Low-Depth Genotyping-by-Sequencing for Genomic Prediction Among Multi-Parental Family Pools and Single Plants in Perennial Ryegrass (Lolium perenne L.)
Source: Front Plant Sci. 2018 Mar 21;9:369. doi: 10.3389/fpls.2018.00369 (PMC5871745; doi:10.3389/fpls.2018.00369)
Supplement: Supplementary file 1 [file DataSheet1.docx]

# Appendix I: Variance of F_2_-family means

L. Janss

Center for Quantitative Genetics and Genomics

Aarhus University, Denmark

We derived the variance of means for F_2_ families, based on the classical quantitative genetic model using pedigree-based relationships (Hill and Mackay, 2004). We only show the case of F_2_ families from two grandparents.

If we let the polygenic value of two grandparents be $u_{11}$ and $u_{12}$ and we assume that the grandparents are unrelated and not inbred, then:

$$var\left( u_{11} \right)=var \left( u_{12} \right)= \sigma_{a}^{2}$$

$$cov(u_{11},u_{12})= 0$$

where $\sigma_{a}^{2}$ is the polygenic variance.

The polygenic values of F_1_ progeny can then be expressed as:

$$u_{2j}=\frac{1}{2}u_{11}+ \frac{1}{2}u_{12}+ \emptyset_{2j}$$

For *j=1, 2, …, J*, where *J* is the number of F_1_ progeny and $\emptyset_{2j}$ is the Mendelian sampling terms for F_1_ progeny.

When grandparents are not inbred:

$$var\left( \emptyset_{2j} \right)= \frac{1}{2}\sigma_{a}^{2}$$

The F_2_ progenies are created from random cross-pollination between F_1_ individual single plants (SP). We assume here that selfing is not possible, which implies that every F_2_ SP must be derived from two different F_1_ SPs. The polygenic values in the F_2_ SPs can then be expressed as:

$u_{3k}= \frac{1}{2} u_{2m}+ \frac{1}{2} u_{2m^{*}}+ \emptyset_{3k}$

for *k* = 1, 2, …, *K*, where *K* is the number of F_2_ SP, *m* and *m** are two different F_1_ SPs (), and $\emptyset_{3k}$ is the Mendelian sampling terms for F_2_ progeny. Because Mendelian sampling variance only depends on inbreeding status of the parents, and F_1_ SPs are not inbred when grandparents are not related, then,

$$var\left( \emptyset_{3k} \right)= \frac{1}{2}\sigma_{a}^{2}$$

However, if the F1 progeny are all full sibs with a co-ancestry of, then

$$cov\left( u_{2m}+ u_{2m^{*}} \right)= \frac{1}{2}\sigma_{a}^{2}$$

From the prior equations we can derive the variance in F_2_ single plants as:

$$var\left( u_{3k} \right) = \frac{1}{4} var \left( u_{2m} \right)+ \frac{1}{4} var \left( u_{2m^{*}} \right)+2 \frac{1}{4} cov\left( u_{2m}, u_{2m^{*}} \right)+var\left( \emptyset_{3k} \right)$$

$$= {\frac{1}{4} \sigma}_{a}^{2}+ {\frac{1}{4} \sigma}_{a}^{2}+{\frac{1}{4} \sigma}_{a}^{2}+{\frac{1}{2} \sigma}_{a}^{2}$$

$$=1{\frac{1}{4} \sigma}_{a}^{2}$$

This is a straightforward result, showing that variance in F_2_ single plants is increased by because their parents (F_1_'s) were full sibs.

To derive the genetic variance for F2 family means, we denote the mean polygenic value of an F_2_ family as $\bar{u}_{3}$ where

$$\bar{u}_{3}= \frac{1}{K} \sum u_{3k}$$

and the sum is over the *k = 1, 2, …, K,* F_2_ progeny. First, we rewrite this sum by rewriting the polygenic values of F_2_ single plants, using F_1_ polygenic values, and then rewrite the F_1_ polygenic values using grandparental polygenic values so that:

$$\sum u_{3k}= \sum\frac{1}{2}u_{2m}+ \frac{1}{2}u_{2m^{*}}+ \emptyset_{3k}$$

$$=\sum\frac{1}{2} \left( \frac{1}{2}u_{11}+ \frac{1}{2}u_{22}+ \emptyset_{2m} \right)+ \frac{1}{2} \left( \frac{1}{2}u_{11}+ \frac{1}{2}u_{22}+ \emptyset_{2m^{*}} \right)+\emptyset_{3k}$$

$$=\sum\frac{1}{2}u_{11}+ \frac{1}{2}u_{12}+ \frac{1}{2} \emptyset_{2m}+ \frac{1}{2} \emptyset_{2m^{*}}+ \emptyset_{3k}$$

Subsequently, we need to compute the variance of this sum. To do so, first this sum is split in parts, as long as the parts are independent. All Mendelian sampling terms are independent of grandparental breeding values and are independent of all other Mendelian sampling terms. However, the $\emptyset_{2m}$ and $\emptyset_{2m^{*}}$ are Mendelian sampling terms of F_1_ parents present in the F_2_ single plant breeding values, which may have duplicates for F_2_ single plants that descend from the same F_1_ parent. Therefore we rewrite this sum as:

$$\sum u_{3k}=\frac{1}{2} Ku_{11}+\frac{1}{2} Ku_{12}+ \frac{1}{2}\sum\left( \emptyset_{2m} + \emptyset_{2m^{*}} \right)+ \sum\emptyset_{3k}$$

Now, it follows that the variance of the mean breeding value of F_2_ families is:

$$var\left( \bar{u}_{3} \right)= \frac{1}{K^{2}} var \left( \frac{1}{2}Ku_{11} \right)+ \frac{1}{K^{2}} var \left( \frac{1}{2}Ku_{12} \right)+ \frac{1}{K^{2}} var\left( \frac{1}{2} \sum\left( \emptyset_{2m} + \emptyset_{2m^{*}} \right) \right)+ \frac{1}{K^{2}} var(\sum\emptyset_{3k})$$

$$= \frac{1}{4} var\left( u_{11} \right)+ \frac{1}{4} var\left( u_{12} \right)+ \frac{1}{K^{2}} \frac{1}{4} var \left( \sum\left( \emptyset_{2m} + \emptyset_{2m^{*}} \right) \right)+ \frac{1}{K^{2}} K var(\emptyset_{3k})$$

To compute $var \left( \sum\left( \emptyset_{2m} + \emptyset_{2m^{*}} \right) \right)$ we first note that this is a sum over 2*K* F_1_ Mendelian sampling terms: the variance of this sum is 2*K* times the variance of an F_1_ single plant Mendelian sampling term $var(\emptyset_{2j})$, plus 4*K*^2^-2*K* times the covariance between two randomly-sampled F_1_ single plant Mendelian sampling terms. This covariance is $var(\emptyset_{2j})$ when the Mendelian sampling terms are identical, which happens with a probability 1/*J*. Thus, we evaluate the variance of this sum as: $2K var\left( \emptyset_{2j} \right)+\left( 4K^{2}-2K \right) \frac{1}{J} var(\emptyset_{2j})$. The final expression for the variance of F_2_ family means is then:

$var\left( \bar{u}_{3} \right)= \frac{1}{2}\sigma_{a}^{2}+\left( \frac{1}{2K}+\frac{1}{J}-\frac{1}{2KJ} \right)var$($\emptyset_{2j})+ \frac{1}{K} var(\emptyset_{3k})$

This expression shows no effect from inbreeding within the F_2_ on the variance of F_2_-family means, but it shows effects of finite sample size in F_1_ and F_2_ families. However, even for quite modest sample size, say *K* and *J* > 50, the effects of finite sample size are already small, and the variance of F_2_-family means is simply half the genetic variance. This result was also found in the simulation study in the main text. Note that the above result is derived assuming no relationship between the grandparents; accounting for such a relationship would increase the variance of F_2_-family means.
